# Reduced hyaluronan cross-linking induces breast cancer malignancy in a CAF-dependent manner

**DOI:** 10.1038/s41419-021-03875-6

**Published:** 2021-06-07

**Authors:** Guoliang Zhang, Yiqing He, Yiwen Liu, Yan Du, Cuixia Yang, Feng Gao

**Affiliations:** 1 Department of Molecular Biology, Shanghai Jiao Tong University Affiliated Sixth People’s Hospital, 600 Yishan Road, 200233 Shanghai, China; 2 Department of Clinical Laboratory, Shanghai Jiao Tong University Affiliated Sixth People’s Hospital, 600 Yishan Road, 200233 Shanghai, China

**Keywords:** Breast cancer, Cancer microenvironment

## Abstract

Hyaluronan (HA) cross-linking is a conformational state of HA, a covalent complex between HA and heavy chains (HCs) from inter-α-trypsin inhibitor (I-α-I) mediated by tumor necrosis factor-induced protein 6 (TSG6). Cross-linked HA has been identified as a protective factor in physiological and inflammatory conditions. However, the state of HA cross-linking in tumor microenvironment has not been fully elucidated. As a major constituent of the extracellular matrix (ECM), HA is mainly synthesized by cancer-associated fibroblasts (CAFs). Our study aimed to clarify the role of HA cross-linking in breast cancer malignancy. Compared to normal mammary gland tissues, cross-linked HA levels were significantly decreased in breast cancer and associated with tumor malignancy. When NFbs were activated into CAFs, the levels of cross-linked HA and TSG6 were both suppressed. Through upregulating TSG6, CAFs restored the high level of cross-linked HA and significantly inhibited breast cancer malignancy, whereas NFbs promoted the malignancy when the cross-linked HA level was reduced. Furthermore, the inhibitory role of HA cross-linking in tumor malignancy was directly verified using the synthesized HA-HC complex. Collectively, our study found that the deficiency of cross-linked HA induced breast cancer malignancy in a CAF-dependent manner, suggesting that recovering HA cross-linking may be a potential therapeutic strategy.

## Introduction

During malignancy, tumor cells must establish a favorable microenvironment or niche that will sustain their growth. Cancer-associated fibroblasts (CAFs) are the most abundant mesenchymal cells in the tumor microenvironment^1^. CAFs not only promote tumor malignancy but also serve as a marker for cancer diagnosis, treatment, and prognosis^2–4^. When normal fibroblasts (NFbs) are activated into CAFs, the cytokines secreted and extracellular matrix (ECM) around were altered dramatically^5^.

The pathological remodeling of ECM affects the proliferation, survival, and metastasis of tumor cells^6,7^. Hyaluronan (HA), a linear polysaccharide composed of repeating disaccharide units of D-glucuronic acid and *N*-acetyl-d-glucosamine^8^, is the major constituent of ECM. HA is synthesized by three kinds of HA synthases on the plasma membrane and can be secreted into the surrounding microenvironment. Under physiological conditions, HA is immunologically quiescent and can maintain cell stability. In the tumor microenvironment, the synthesis and degradation of HA both increase, resulting in the abnormal accumulation of HA fragments. Most current studies focus on the role of HA with different molecular weights (MWs) in tumor malignancy^9^. For example, low MW HA (LMW-HA) has been reported to possess many pro-tumor activities that are not exerted by high MW HA (HMW-HA). Studies on naked mole rats have found that ultra-high MW HA has an antitumor effect^10^. Through binding to receptors, such as CD44, lymphatic vessel endothelial receptor 1, and receptor for HA-mediated motility, LMW-HA can induce angiogenesis and lymphangiogenesis, accelerate cancer cell migration, and contribute to the expression of matrix metalloproteinases^11–14^. However, whether there is a conformational change of HA in tumor microenvironment has not been well described.

Actually, the HA matrix can be modified by dynamic patterns of hyaladherins, which can change the conformation of HA and expand the repertoire of HA interactions with ECM components. One of the best examples of HA conformational changes is cross-linking. HA cross-linking has been well characterized as the covalent complex formed between HA and heavy chains (HCs) from inter-α-trypsin inhibitor (I-α-I), which is induced by tumor necrosis factor-induced protein 6 (TSG6)^15–17^. TSG-6 can also mediate the cross-linking of HA itself but is impaired in the presence of I-α-I^18^. In TSG6-deficient mice, the formation of covalent complexes between HA and HCs was impaired, making cumulus cells unable to assemble HA-rich ECM, resulting in female mice infertility^19^. HA cross-linking was also found in inflammatory diseases^20,21^. The cross-linked HA in synovial fluid can increase its viscosity, reduce HA loss, and resist the adverse effects of HA-degraded fragments in arthritis^21^. In addition, the cross-linked HA can be used as a “water tank” for oxygen radicals to inhibit inflammation^22^. The above studies show that cross-linked HA can maintain normal fertility and has anti-inflammatory effects. However, the state of HA cross-linking in cancer progression, particularly in tumor microenvironment, is unknown. Given the protective role of cross-linked HA in physiological and inflammatory processes, we propose that HA cross-linking might become deficient during tumor malignancy.

It is well known that HA levels are usually increased in many solid tumors, such as colorectal, prostate, and breast cancer^23–25^. A clinical survey has proved a close correlation between high-stromal HA enrichment and poor survival in breast cancer patients^26,27^. In tumor microenvironment, CAFs are a major source of ECM in “cancerized” stroma that impact tumor initiation and progression. Suppressing the HA synthesis in CAFs can inhibit the malignancy of several solid cancers^28–31^. Therefore, CAFs are critical for studying the role of HA cross-linking in breast cancer malignancy.

In this study, the state of HA cross-linking was first clarified in the breast cancer microenvironment. Next, the change of cross-linked HA levels was observed when NFbs were activated into CAFs. Then the effects of HA cross-linking on tumor malignancy were determined by co-culturing tumor cells with NFbs or CAFs with different levels of cross-linked HA, as well as using the synthesized cross-linked HA. We found that HA cross-linking was deficient in the breast cancer microenvironment due to TSG6 downregulation in CAFs. When cross-linked HA was restored in CAFs, the breast cancer malignancy was significantly suppressed.

## Materials and methods

### Patients

Patients diagnosed with breast cancer (*n* = 16) were enrolled. Patients who received chemotherapy or radiotherapy before surgery were excluded. Details concerning clinical characteristics are provided in Table S1. Tumors were staged according to the tumor–node–metastasis staging system^32^.

### Mouse model and cell culture

The MMTV-PyMT (polyomavirus middle T antigen) mouse model was applied for the widely accepted reason that it can mimic several aspects of human breast cancer, including initiation, histological and molecular progression, metastasis, and immunotherapies. Despite not being a human oncogene, PyMT imitates the signaling of receptor tyrosine kinases, which are commonly activated in human breast cancer^33^.

Primary MMTV tumor cells were isolated from breast cancer tissues of 12–14-week MMTV-PyMT mice. The tumors were isolated and cut into small pieces. After digestion using a mouse tumor disassociation kit (Miltenyi Biotec, Bergisch Gladbach, Germany) according to the manufacturer’s instructions, single cells were obtained. Tumor cells were defined as CD45^−^/EpCAM^+^ cells and purified by a fluorescence-activated cell sorting instrument. NFbs from mammary glands of FVB mice and CAFs from breast cancer tissues of MMTV-PyMT mice were isolated as previously reported^34^. All NFbs and CAFs were used for experiments at P1–P5. Different kinds of fibroblasts were cultured in Dulbecco’s modified Eagle’s medium/F12 medium supplemented with 10% fetal bovine serum (FBS), 1% penicillin/streptomycin (100×), 5 μg/ml insulin, 5 ng/ml basic fibroblast growth factor, 1 μg/ml hydrocortisone, and 50 μg/ml ascorbic acid.

### Immunohistochemistry and immunofluorescence

Tissues from patients or mice were fixed, embedded in paraffin, and cut at 5 μm thickness. The sections were dewaxed, hydrated, processed with antigen retrieval and inhibition of endogenous peroxidase, and blocked. For immunohistochemistry, the sections were covered with anti-Ki67 antibody (1:800, Abcam, Cambridge, MA, USA) and incubated overnight at 4 °C. Then the sections were immersed with secondary antibody, followed by streptavidin-ABC, developed with DAB solution, and counterstained using hematoxylin. For immunofluorescence, the sections were first incubated with anti-vimentin (1:500, Abcam), anti-TSG6 (1:100, R&D, Minneapolis, MN, USA), anti-HC1(1:200, Invitrogen, Carlsbad, CA, USA), or anti-HC2 (1:200, Invitrogen) antibodies overnight at 4 °C. Then the sections were immersed with corresponding fluorescent secondary antibodies labeled with Alexa Fluor 488 for 1 h at room temperature. After incubation with biotinylated hyaluronate-binding protein (HABP, 5 μg/ml, Merck, Darmstadt, Germany) overnight at 4 °C, the sections were immersed with Alexa Fluor^®^ 647-conjugated streptavidin (1:800, Yeasen Biotech, Shanghai, China). Finally, the slides were mounted using an antifade mounting medium with 4,6-diamidino-2-phenylindole (DAPI; Abcam) and photographed with a confocal microscope (Nikon, Japan).

For Ki67 staining, MMTV tumor cells were fixed by 4% paraformaldehyde. After permeabilization and blocking, cells were incubated with anti-Ki67 antibody (1:800, Abcam) overnight at 4 °C. Alexa Fluor^®^ 594-conjugated secondary antibody (1:800, Abcam) was applied and DAPI was used to stain nuclei. For HA staining, NFbs and CAFs were seeded in 24-well cell culture plate with a glass bottom (6 × 10^4^ cells per well) and cultured for 48 h. Cells were fixed by AAF fixative (95% ethyl alcohol 34 ml + glacial acetic acid 2 ml + formalin 4 ml) at −20 °C for 20 min. After blocking and incubation with HABP (5 μg/ml) overnight at 4 °C, Alexa Fluor^®^ 488-conjugated streptavidin was applied at 1:800. Cells were observed under a confocal microscope after staining with DAPI.

### Lentiviral infection

Lenti-shTSG6 (mouse) and control viruses were obtained from Santa Cruz (Dallas, TX, USA). Lenti-TSG6 (mouse) and control viruses were acquired from GeneCopoeia (Rockville, MD, USA). Lenti-shTSG6 and corresponding viruses were transfected into NFbs with polybrene (5 μg/ml), whereas Lenti-TSG6 and control viruses were transfected into CAFs. After 24 h, the cells were selected using puromycin for another 48 h and used for different kinds of experiments.

### Western blot

Cells were washed with cold phosphate-buffered saline (PBS) and lysed in RIPA lysis buffer. Then proteins were separated via sodium dodecyl sulfate-polyacrylamide gel electrophoresis (10%) gels and transferred to polyvinylidene difluoride membranes. The membranes were incubated with blocking buffer, followed by primary antibodies against vimentin (1:1000, Abcam), alpha-smooth muscle actin (α-SMA; 1:1000, Sigma, St. Louis, USA), TSG6 (1:100, R&D), I-α-I (1:1000, Dako, Carpinteria, CA, USA), and glyceraldehyde 3-phosphate dehydrogenase (GAPDH; 1:1000, CST) as well as horseradish peroxidase (HRP)-labeled secondary antibodies. ECL method was used to detect the specific bands.

### Real-time PCR

Total RNA was isolated from NFbs and CAFs using TRIzol reagent (Takara, Japan). After quantification using a NanoDrop 2000 spectrophotometer, purified total RNA was reverse-transcribed, and quantitative real-time PCR was conducted by an ABI 7500 instrument with SYBR Premix Ex Taq™ (Takara) according to the manufacturer’s instructions. The primers used were mouse *TSG6*, 5′-GATACAAGCTCACCTACGCCGAAG-3′ (forward) and 5′-GCCATCCATCCAGCAGCACAG-3′ (reverse); and *GAPDH*, 5′-CATCAC TGCCACCCAGAAGACTG-3′ (forward) and 5′-ATGCCAGTGAGCTTCCCGT TCAG-3′ (reverse).

### Tumor–stromal assay (TSA)

In TSA, 12-well cell culture plates with a glass bottom and press-to-seal silicon isolators with round wells (2 mm diameter, Sigma) were used. The culture plate was coated with collagen overnight, and isolators were put into glass wells. After staining with a cell tracker, MMTV tumor cells were seeded into the round wells of isolators (3000 cells per well) and incubated for 1 h. After incubation overnight in 1 ml complete medium, isolators were removed. Then 5 × 10^4^ fibroblasts were seeded and incubated for 30 min. The wells were washed to remove the fibroblasts that landed on the tumor cell islands. After 72 h, Ki67 was stained.

### Apoptosis assay

The apoptosis was determined using TACS® 2TdT-DAB Apoptosis Detection Kit (Trevigen, Gaithersburg, USA) as per the manufacturer’s instruction. MMTV tumor cells were seeded into 96-well plates and stimulated with or without 50% conditioned media (CM) from different fibroblasts for 72 h. Samples were fixed by 4% paraformaldehyde and covered with 50 μl of Cytonin^TM^ for 30 min. After immersing in quenching solution for 5 min and washing by PBS, samples were incubated in labeling buffer for 5 min. Next, samples were covered with 50 μl of labeling reaction mix and incubated for 60 min at 37 °C. After stopped by the TdT stop buffer, samples were covered with 50 μl of Strep-HRP solution for 10 min at 37 °C. Then samples were incubated in DAB solution for 3 min and counterstained by 1% methyl green for 1 min. For the quantification of apoptotic cells, five randomly selected fields (×400 magnification) were counted.

### Colony formation assay

MMTV tumor cells were seeded at 1000 cells per well in the 12-well culture plates and stimulated with 50% CM of different fibroblasts. After incubation at 37 °C for 10 days, cells were fixed with 4% paraformaldehyde and stained with 0.1% crystal violet solution.

### Migration and invasion assay

The migration and invasion were conducted using 8 μm transwell chambers (Corning, Cambridge, USA) and 8 μm matrigel invasion chambers (BD Biosciences), respectively. In all, 5 × 10^4^ tumor cells were seeded for migration and 1 × 10^5^ cells for invasion. The upper chambers were filled with 250 μl serum-free medium or 100% CM from fibroblasts, whereas the lower chambers were filled with 500 μl medium supplemented with 10% FBS. After 24 h, invaded cells were stained with crystal violet and quantified under a microscope by counting them in five random fields (×100 magnification).

For the direct co-culture, 5 × 10^4^ tumor cells pre-stained with cell tracker (green, Invitrogen) were mixed with or without different fibroblasts (5 × 10^4^) and seeded in the upper chambers of migration or invasion assay. After incubation for 24 h, cells invading into the bottom surface of the inserts were observed under a fluorescence microscope.

### HA electrophoresis

CM of fibroblasts was concentrated and digested at 50 °C overnight using proteinase K (Sigma). After the proteinase K was inhibited by pefabloc SC (Sigma), benzonase endonuclease (Sigma) was added to remove nucleic acid. Total polysaccharides were extracted using phenol/chloroform extraction followed by EtOH precipitation. Finally, a corresponding volume of 10 mM Tris-HCl (pH 8.5) was added to dissolve the pellet. The size distribution of HA was analyzed by agarose gel electrophoresis. The HA samples were loaded on a 0.8% agarose gel and electrophoresed. The gel was stained with 0.005% Stains-All (Sigma) overnight in the dark. Then the gel was placed in distilled H_2_O under ambient light to destain and photographed.

### HA-HC complex synthesis

HA-HC complex was synthesized as reported before^35^. HA (H5388, Sigma), recombinant mouse TSG-6 protein (R&D), and mouse serum (isolated from female FVB mouse) were used. The reaction volume of HA-HCa was 250 μl, containing 50 μl HA (2 mg/ml, Sigma), 100 μl mouse serum, 80 μl TSG6 (5 μg/ml, R&D), and 20 μl PBS. The volumes were altered correspondingly in the groups of HA, HA + TSG6, HA + I-α-I, and HA-HCb. The mixtures were incubated at 37 °C for 24 h and stopped by adding 5 μl EDTA solution (0.5 M, pH 8.0). After the reaction, the products were incubated with or without 20 units/ml hyaluronidase at 37 °C for 1 h. Then the samples were combined with loading buffer and boiled to denaturation. Western blot was used to confirm the existence of HA-HC complex. After dilution (1:8) with culture media, HA-HC complex was used to stimulate MMTV tumor cells.

### Animal experiment

Female athymic BALB/c nude mice (5–6 weeks old) were used for animal studies. MMTV tumor cells (1 × 10^5^) alone or admixed with different fibroblasts (3 × 10^5^) were orthotopically injected into the mammary fat pads of mice (*n* = 5 per group). Cells were resuspended in cold 50% Growth Factor Reduced Matrigel (BD Biosciences) before injection. Mice were randomly allocated to different experimental groups and processed. The investigator was not blinded to the group allocation during the experiment. Tumors were measured with calipers every 3–4 days, and volumes were calculated using the formula “width^2^ × length × 0.52.” Mice were sacrificed when the tumor diameter exceeded 1 cm. Then samples were isolated, photographed, fixed, and embedded by paraffin for immunohistochemistry and immunofluorescence.

### Statistical analysis

Statistical analyses between two groups were performed using Student’s *t* test or Mann–Whitney rank-sum test (where appropriate). The variance between the groups that are being statistically compared is similar. SPSS 19.0 statistical software (SPSS, San Diego, CA, USA) was used to analyze our data. In all cases, *p* < 0.05 was considered to be significant.

## Results

### HA cross-linking was decreased in breast cancer tissues and associated with tumor stage

**Fig. 1:**
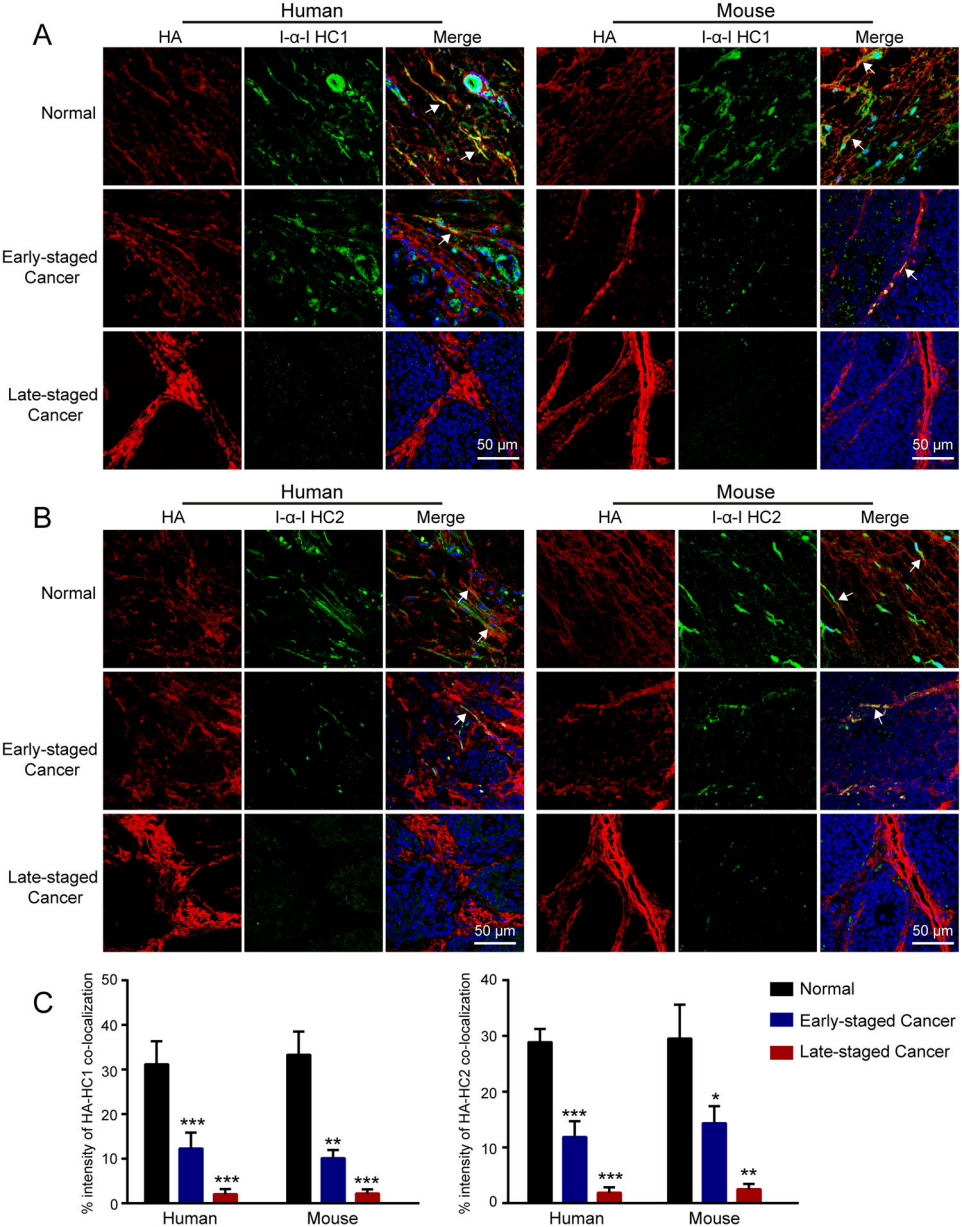
HA cross-linking was decreased in breast cancer tissues and associated with tumor stage. **A** Immunofluorescence staining of HA (red) and I-α-I HC1 (green) in cancer tissues and corresponding adjacent normal tissues from patients with early (stage I, *n* = 7) or late (stage III, *n* = 9) staged breast cancer, normal mammary gland tissues from FVB mice, and cancer tissues from MMTV-PyMT mice with early (week 10) or late (week 16) staged breast cancer. **B** Immunofluorescence staining of HA (red) and I-α-I HC2 (green) in corresponding tissues. The white arrowheads refer to the co-localization of HA and I-α-I, which means HA cross-linking. HA and I-α-I HCs were both localized within and outside of cells. **C** The intensity ratio of HA co-localized with I-α-I HCs to total HA was used to determine the levels of HA-HC complexes. The intensity percentage of HA-HCs co-localization was acquired by the Image J software. Error bars show mean ± SD values. Statistical analysis was performed using Student’s *t* test. *p < 0.05, **p < 0.01, ***p < 0.001.

HCs of I-α-I can be transferred to HA, forming the HA-HC complex, which is called the cross-linked HA. Therefore, the co-localization of HA and I-α-I HCs is usually used to determine the existence of HA cross-linking in tissues^15,16,36^. In our study, HA and I-α-I HCs were simultaneously stained using immunofluorescence to verify the expression of cross-linked HA in tissues from breast cancer patients. In normal breast tissues, HA was co-localized with I-α-I HC1 or I-α-I HC2, suggesting that HA was covalently modified with HCs of I-α-I to form HA-HC complexes. In contrast, both HA-HC1 and HA-HC2 complexes were significantly decreased in cancer tissues from patients with stage I breast cancer and were almost disappeared in stage III breast cancer (Fig. 1).

We also confirm the result using normal mammary gland tissues from wild-type (FVB) mice and cancer tissues from MMTV-PyMT mice with early (week 10) or late (week 16) stage breast cancer. As shown by immunofluorescence, the levels of HA-HC1 and HA-HC2 complexes were markedly decreased in breast cancer tissues from MMTV-PyMT mice and negatively associated with breast cancer stages (Fig. 1). Moreover, immunoblotting was used to further clarify the difference of HA-HC complex in mouse normal and breast cancer tissues. As shown in Fig. S1A, the level of HA-HC complex was extensively decreased in cancer tissues.

### CAFs were responsible for HA cross-linking deficiency in breast cancer

**Fig. 2:**
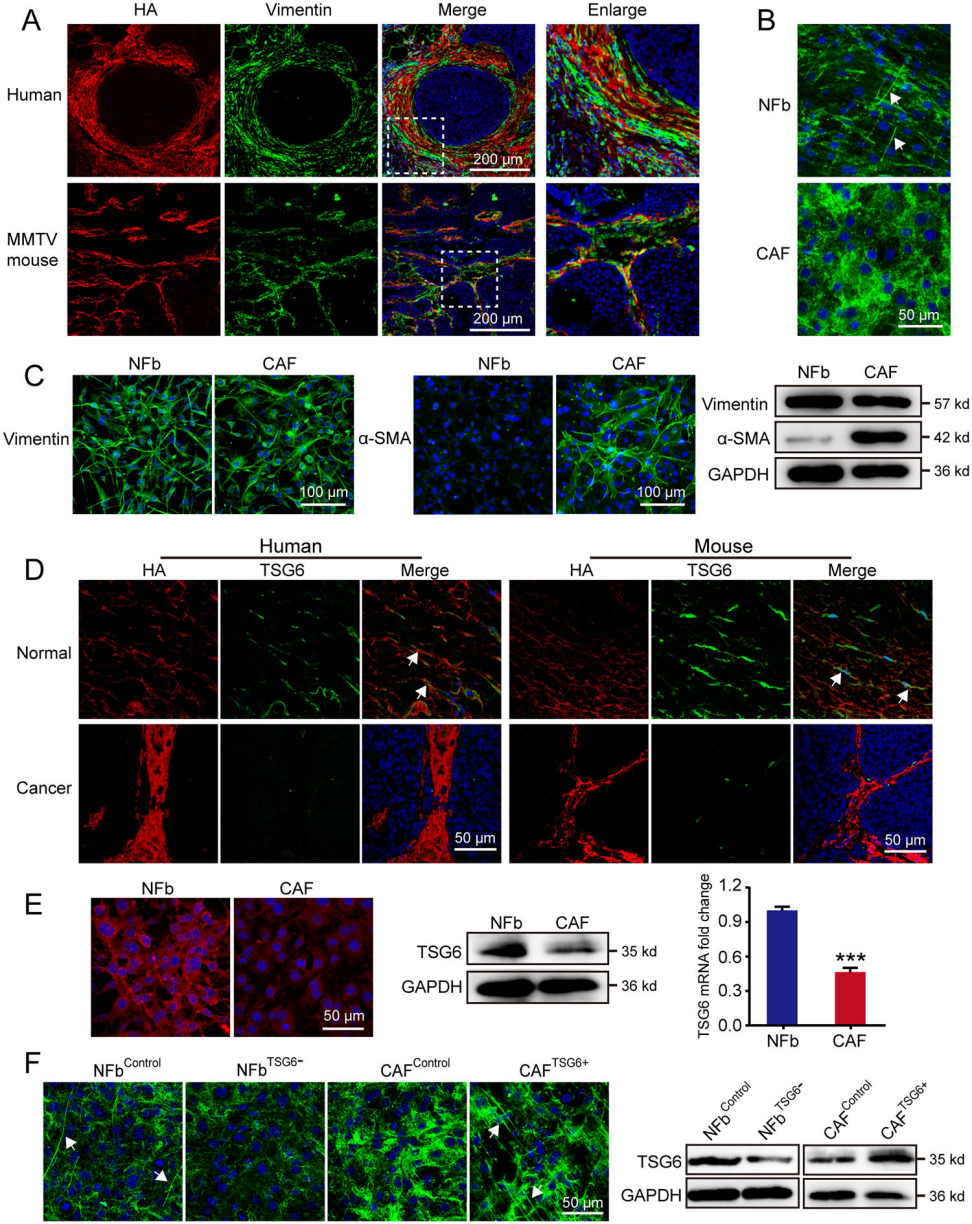
Cancer-associated fibroblasts were responsible for HA cross-linking deficiency in breast cancer. **A** HA (red) and vimentin (green) staining in breast cancer tissues from patients and MMTV-PyMT mice. HA and vimentin were both mainly localized in the stroma of cancer tissues. Vimentin was expressed within cells, whereas HA was found within and outside of cells. **B** Normal fibroblasts (NFbs) and cancer-associated fibroblasts (CAFs) were derived from normal breast tissues of FVB mice and breast cancer tissues of MMTV-PyMT mice, respectively. Immunofluorescence staining of HA was conducted in NFbs and CAFs. The cable-like structures, as pointed by the white arrows, are the cross-linked HA. **C** Vimentin and α-SMA were detected in NFbs and CAFs using immunofluorescence and western blot. **D** HA and TSG6 were stained in normal and breast cancer tissues from patients and mice. White arrows point to the co-localization of HA and TSG6. TSG6 was localized within and outside of cells. **E** The expression levels of TSG6 in NFbs and CAFs were determined by immunofluorescence, western blot, and real-time PCR. Error bars show mean ± SD values. Statistical analysis was performed using Student’s *t* test. ***p < 0.001. **F** NFbs were infected with Lenti-Control or Lenti-shTSG6 viruses (NFb^Control^ and NFb^TSG6−^). Meanwhile, CAFs were infected with Lenti-Control or Lenti-TSG6 viruses (CAF^Control^ and CAF^TSG6+^). The expression levels of TSG6 were confirmed by western blot. Then HA was stained to observe HA cross-linking, as indicated by the white arrows.

To clarify which kind of cells are responsible for the deficiency of HA cross-linking in breast cancer, we simultaneously determined the expression of HA and vimentin using immunofluorescence. As shown in Fig. 2A and Fig. S1B, the distribution pattern of HA was the same as vimentin, suggesting that CAFs are the main source of HA. Then we isolated NFbs and CAFs from mammary glands of FVB mice and breast cancer tissues of MMTV-PyMT mice, respectively. NFbs and CAFs were both positive for vimentin, whereas α-SMA was only significantly increased in CAFs (Fig. 2C), indicating that NFbs and CAFs were successfully isolated and cultured. Importantly, we found that HA could form cable-like structures in NFbs, which was the cross-linked HA. However, the cross-linked HA was deficient in CAFs (Fig. 2B). Furthermore, NFbs and CAFs were isolated and cultured from surgical specimens of breast cancer patients. As shown in Fig. S2A, HA cross-linking was also disappeared in CAFs.

TSG6 has been implicated as a critical regulator of HA cross-linking in inflammatory diseases and can be expressed in macrophages stimulated with lipopolysaccharide^37^. Next, we determine whether TSG6 contributes to the deficiency of cross-linked HA in breast cancer. In early-staged breast cancer tissues, TSG6 was mainly derived from CAFs, not macrophages (Fig. S3). The level of TSG6 was significantly decreased in late-staged breast cancer tissues (Fig. 2D). Moreover, TSG6 was also considerably downregulated in CAFs (Fig. 2E). Then we validated the role of TSG6 in mediating HA cross-linking. When TSG6 was downregulated, the cross-linked HA was markedly reduced in NFbs (Fig. 2F and Fig. S4A). Our rescue experiment indicated that only wild-type TSG6 could restore the HA cross-linking in NFb^TSG6−^, whereas catalytically inactive TSG6 (S28A)^38^ had no effect (Fig. S4B). Meanwhile, the cross-linked HA was significantly increased in CAFs after restoring TSG6 expression (Fig. 2F).

### Disorganization of HA cross-linking in NFbs promoted breast cancer malignancy

**Fig. 3:**
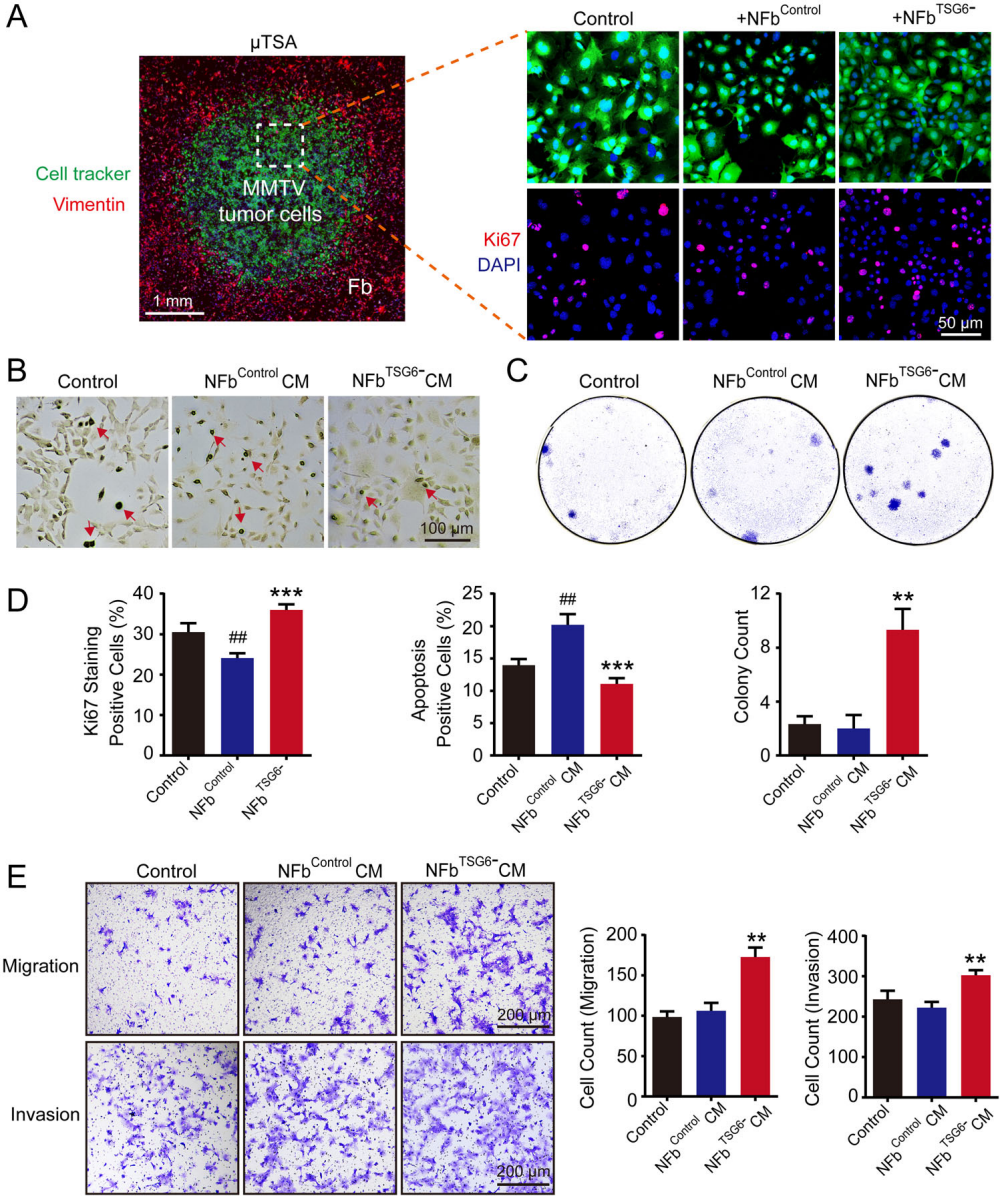
Disorganization of HA cross-linking in normal fibroblasts promoted breast cancer malignancy. **A** Primary tumor cells from MMTV-PyMT mice were stained with cell tracker (green) and then co-cultured with or without corresponding normal fibroblasts (NFb^Control^ and NFb^TSG6−^) in the tumor–stromal assay (TSA). As shown in the left image, tumor cells (green) were in the middle of TSA and surrounded by fibroblasts (red, illustrated by staining vimentin). After 72 h, the proliferation of tumor cells was determined by staining Ki67. The top panel shows the representative images of tumor cells (green) with nuclei (DAPI). The down panel shows the Ki67 expression (red) in corresponding nuclei. **B** After stimulation with 50% conditioned media (CM) of NFb^Control^ or NFb^TSG6−^, the apoptosis of tumor cells was evaluated by the TdT-DAB apoptosis assay. Red arrows indicate the apoptotic tumor cells. **C** The colony formation abilities of tumor cells treated with NFb^Control^ CM or NFb^TSG6−^ CM. **D** The statistical histograms of Ki67 percentage, apoptotic percentage, and colony count of tumor cells in different groups. **E** The migration and invasion abilities of tumor cells. All experiments were repeated three times with similar results. Error bars represent mean ± SD values. Statistical analysis was performed using Student’s *t* test. **p < 0.01, ***p < 0.001 (vs NFb^Control^ or NFb^Control^ CM), ^##^*p* < 0.01 (vs Control).

To explore the role of HA cross-linking in tumor malignancy, we altered the expression of cross-linked HA in fibroblasts by regulating TSG6. First, we downregulated TSG6 expression in NFbs derived from normal mammary glands of FVB mice. NFb^Control^ and NFb^TSG6−^ with different levels of cross-linked HA were separately co-cultured with MMTV tumor cells. The TSA was used to simulate the interaction between tumor cells and stromal fibroblasts. As shown in Fig. S5A and Fig. 3A, the tumor cell islands were surrounded by fibroblasts. In TSA, the Ki67-positive rate of tumor cells was suppressed by NFb^Control^ cells, whereas NFb^TSG6−^ cells with reduced cross-linked HA significantly promoted the proliferation (Fig. 3A, D). Meanwhile, the apoptotic rate of tumor cells treated with 50% CM of NFb^Control^ cells was higher than with tumor cells alone but was significantly decreased when stimulated by NFb^TSG6−^ CM (Fig. 3B, D). Besides, NFb^TSG6−^ CM markedly accelerated the colony formation ability (Fig. 3C, D). For migration and invasion, NFb^TSG6−^ CM with a reduced level of cross-linked HA had a promoting effect on tumor cells compared to NFb^Control^ CM (Fig. 3E). A similar result was obtained when tumor cells stained with a cell tracker were directly co-cultured with NFb^Control^ or NFb^TSG6−^ cells (Fig. S4B).

### The ability of CAFs in promoting breast cancer was inhibited when HA cross-linking was reacquired

**Fig. 4:**
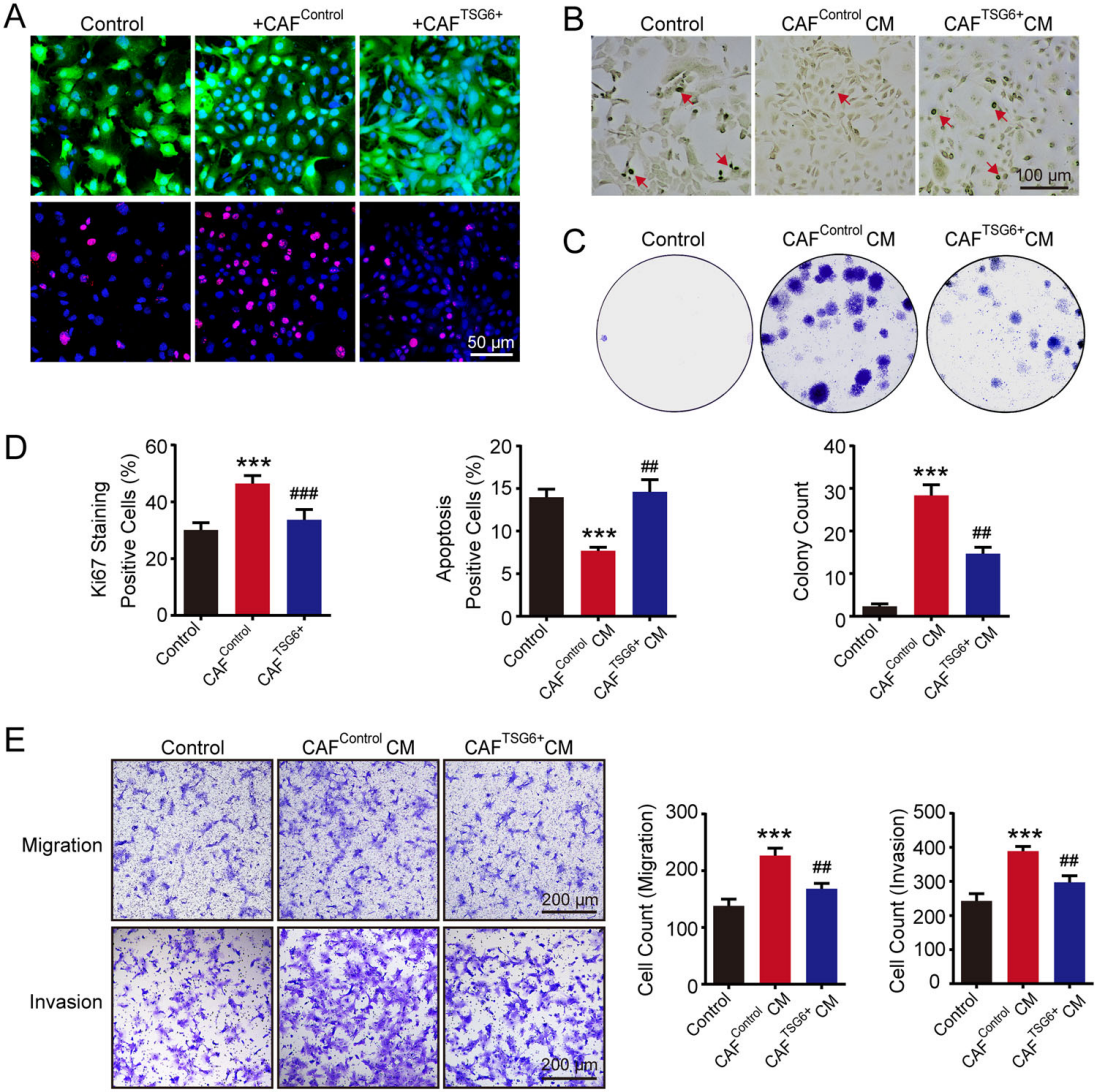
The ability of CAF in promoting breast cancer malignancy was inhibited when HA cross-linking was reacquired. **A** MMTV tumor cells stained with cell tracker (green) were co-cultured with or without corresponding cancer-associated fibroblasts (CAF^Control^ and CAF^TSG6+^) in TSA. After 72 h, the proliferation of tumor cells was determined by staining Ki67. **B** After stimulation with conditioned media (CM) of CAF^Control^ or CAF^TSG6+^, the apoptosis (red arrows) of tumor cells was evaluated. **C** The colony formation abilities of tumor cells treated with CAF^Control^ CM or CAF^TSG6+^ CM. **D** The statistical histograms of Ki67 percentage, apoptotic percentage, and colony count of tumor cells in different groups. **E** The migration and invasion abilities of tumor cells. All experiments were repeated three times with similar results. Error bars represent mean ± SD values. Statistical analysis was performed using Student’s *t* test. ***p < 0.001 (vs Control), ^##^*p* < 0.01, ^###^*p* < 0.001 (vs CAF^Control^ or CAF^Control^ CM).

Next, we tried to verify whether CAFs would affect breast cancer malignancy when HA cross-linking was restored. After overexpression of TSG6, the level of cross-linked HA in CAFs derived from breast cancer tissues of MMTV-PyMT mice can be significantly increased (Fig. 2F). Then CAF^Control^ and CAF^TSG6+^ cells were separately co-cultured with tumor cells. As shown in Fig. 4A, D, the Ki67-positive rate in TSA was significantly higher in tumor cells co-cultured with CAF^Control^ cells. However, CAF^TSG6+^ cells with a high level of cross-linked HA markedly suppressed the proliferation. In the apoptosis assay, CAF^Control^ CM significantly inhibited the apoptotic rate, which was not observed in CAF^TSG6+^ CM (Fig. 4B, D). Besides, the acceleration of CAF^TSG6+^ CM to the colony formation of tumor cells was substantially decreased (Fig. 4C, D). When MMTV tumor cells were stimulated with 50% CAF CM or directly co-cultured with CAFs, the migration and invasion abilities were inhibited by CAF^TSG6+^ cells (Fig. 4E and Fig. S5C).

To explore the possible effect of TSG6 on the MW of HA in fibroblasts, HA derived from NFb^Control^, NFb^TSG6−^, CAF^Control^, and CAF^TSG6+^ cells was analyzed using electrophoresis. As shown in Fig. S6A, TSG6 did not alter the MW of HA significantly. In addition, TSG6 had no significant effect on the levels of pro-tumor cytokines transforming growth factor (TGF)-β, interleukin (IL)-6, epidermal growth factor (EGF), and hepatocyte growth factor (HGF) secreted by CAFs (Fig. S6B).

### Cross-linked HA constructed in vitro suppressed breast cancer malignancy

**Fig. 5:**
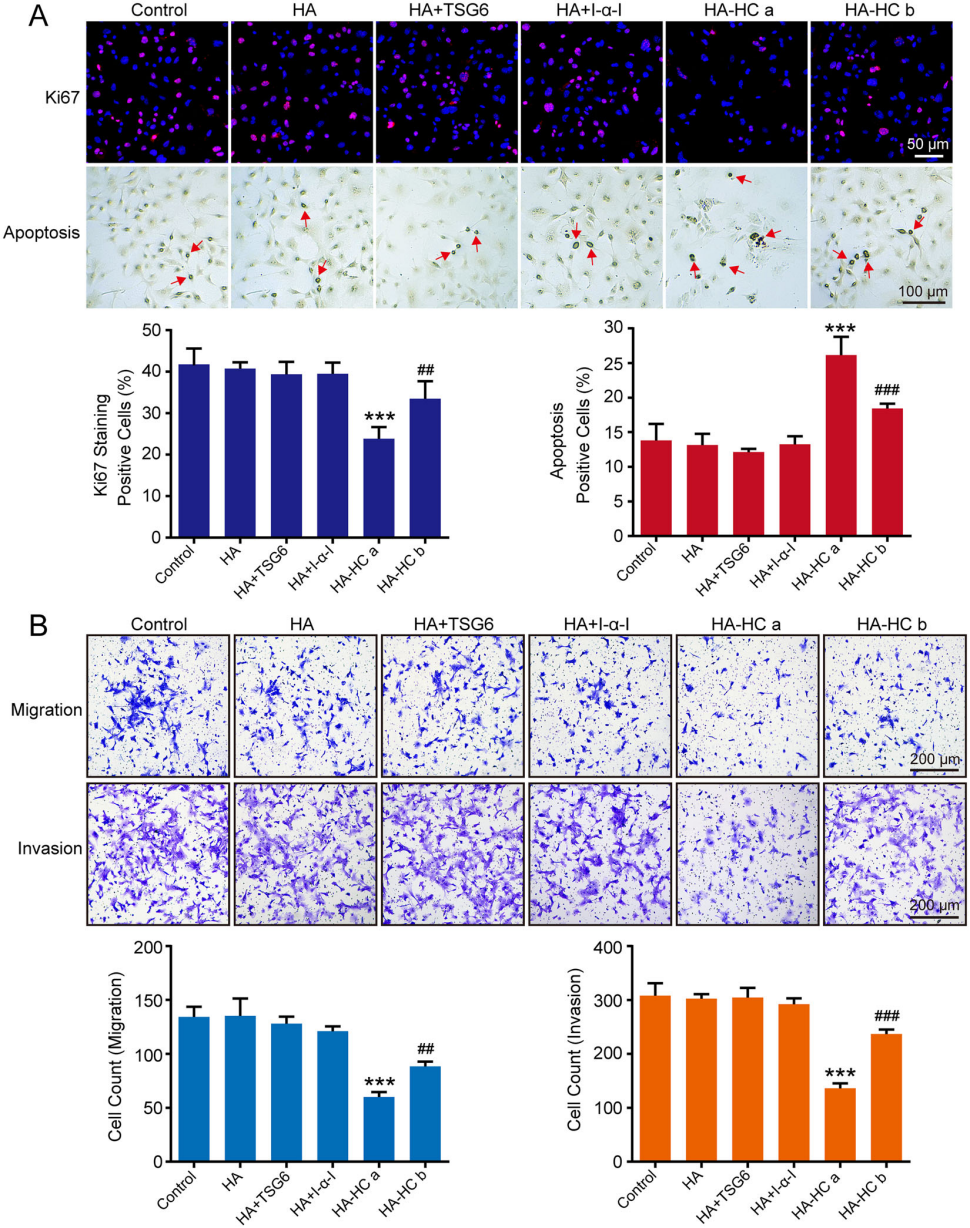
HA cross-linking constructed in vitro suppressed breast cancer malignancy. **A** After treatment with HA, HA + TSG6, HA + I-α-I, HA-HCa (HA + I-α-I + TSG6), or HA-HCb (HA + I-α-I + 1/2 concentrated TSG6), the proliferation and apoptosis of MMTV tumor cells were determined. Red nuclei refer to the Ki67-positive ones, and red arrowheads refer to the apoptotic tumor cells. **B** The migration and invasion abilities of tumor cells in different groups. All experiments were repeated three times with similar results. Error bars represent mean ± SD values. Statistical analysis was performed using Student’s *t* test. ***p < 0.001 (vs Control), ^##^*p* < 0.01, ^###^*p* < 0.001 (vs HA-HCa).

The above results have shown that the different levels of cross-linked HA in fibroblasts can significantly affect the malignancy of breast cancer. To further identify the role of HA cross-linking alone, we next synthesized cross-linked HA (HA-HC complex) in vitro using HMW-HA and evaluated its effects on MMTV tumor cells. The cross-linked HA was confirmed by western blot (Fig. S7). As shown in Fig. 5A, HA-HCa significantly suppressed the Ki67-positive rate and promoted the apoptosis of tumor cells, whereas HA, HA + I-α-I, and HA + TSG6 had no significant effect. Considering the critical role of TSG6 in the formation of HA-HC complex, the concentration of TSG6 in HA-HCb was reduced to half of that used in HA-HCa, which means that the quantity of HA-HC complex in HA-HCb was significantly reduced. As expected, the inhibitory effects on MMTV tumor cells were markedly relieved in the HA-HCb group (Fig. 5A), suggesting that the impact of HA-HC complex was concentration dependent. For migration and invasion, a similar result was observed (Fig. 5B).

Moreover, to determine whether LMW-HA enriched in tumor microenvironment could have cross-linking activity, different kinds of LMW-HA were used to synthesize cross-linked LMW-HA. The result showed that only 200 kDa HA could form the HA-HC complex (Fig. S8A). As shown in Fig. S8B, C, LMW-HA promoted the proliferative and invasive abilities of tumor cells, whereas the pro-tumor effects were not observed in cross-linked LMW-HA, further confirming the suppressive role of HA-HC complex in tumor malignancy.

### HA cross-linking deficiency promoted breast cancer malignancy in vivo

**Fig. 6:**
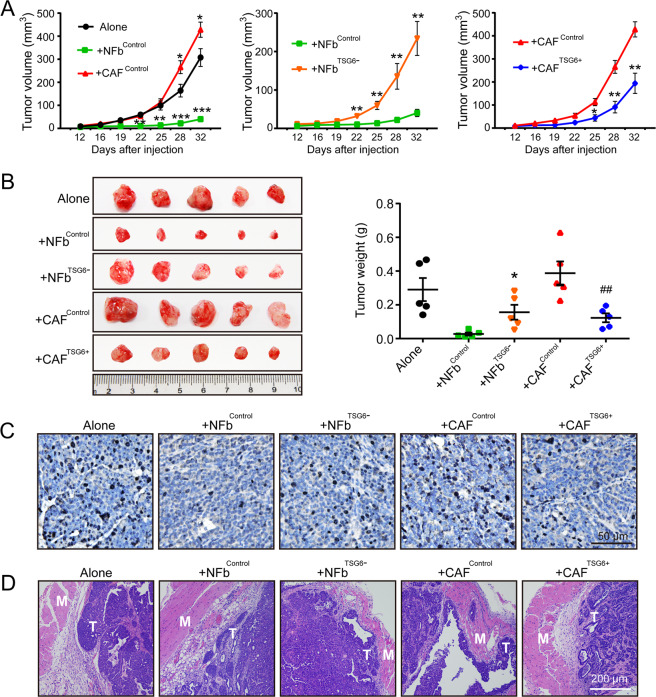
The tumor growth and invasion of MMTV tumor cells in vivo. MMTV tumor cells alone or mixed with NFb^Control^, NFb^TSG6−^, CAF^Control^, and CAF^TSG6+^ cells were injected into mammary fat pads of female nude mice (*n* = 5 each group). **A** Graphs showing the change of tumor volume over time. Error bars represent mean ± SEM values. Statistically significant differences were determined using *t* test. *p < 0.05, **p < 0.01, ***p < 0.001. **B** Tumor images and statistical graph of tumor weight. Error bars represent mean ± SEM values. Statistical analysis was performed using Mann–Whitney test. *p < 0.05 (vs +NFb^Control^), ^##^*p* < 0.01 (vs +CAF^Control^). **C** Ki67 expression in tumor tissues from different groups. Black nuclei indicate Ki67-positive ones. **D** H&E staining of muscle (M) and tumor (T) tissues.

The inhibitory role of HA crossing-linking in the process of breast cancer malignancy has been verified in vitro. Next, MMTV tumor cells alone or mixed with NFb^Control^, NFb^TSG6−^, CAF^Control^, and CAF^TSG6+^ cells were orthotopically injected into mammary fat pads of female nude mice to observe the tumor growth and invasion. As shown in Fig. 6A, CAF^Control^ cells could significantly promote tumor growth, whereas the growth was markedly suppressed by NFb^Control^ cells. In contrast to the NFb^Control^ group, the NFb^TSG6−^ group had much larger tumor volumes. Meanwhile, the tumor volumes in the CAF^TSG6+^ group were significantly smaller than those in the CAF^Control^ group. A similar result was also found in tumor weights (Fig. 6B). As shown in Fig. 6C, the Ki67-positive rates in the NFb^Control^ and CAF^TSG6+^ groups were significantly lower than that in the NFb^TSG6−^ and CAF^Control^ groups, respectively. Besides, we found that MMTV tumor cells in the NFb^TSG6−^ and CAF^Control^ groups were much easier to invade the muscle tissues than those in the NFb^Control^ and CAF^TSG6+^ groups (Fig. 6D).

**Fig. 7:**
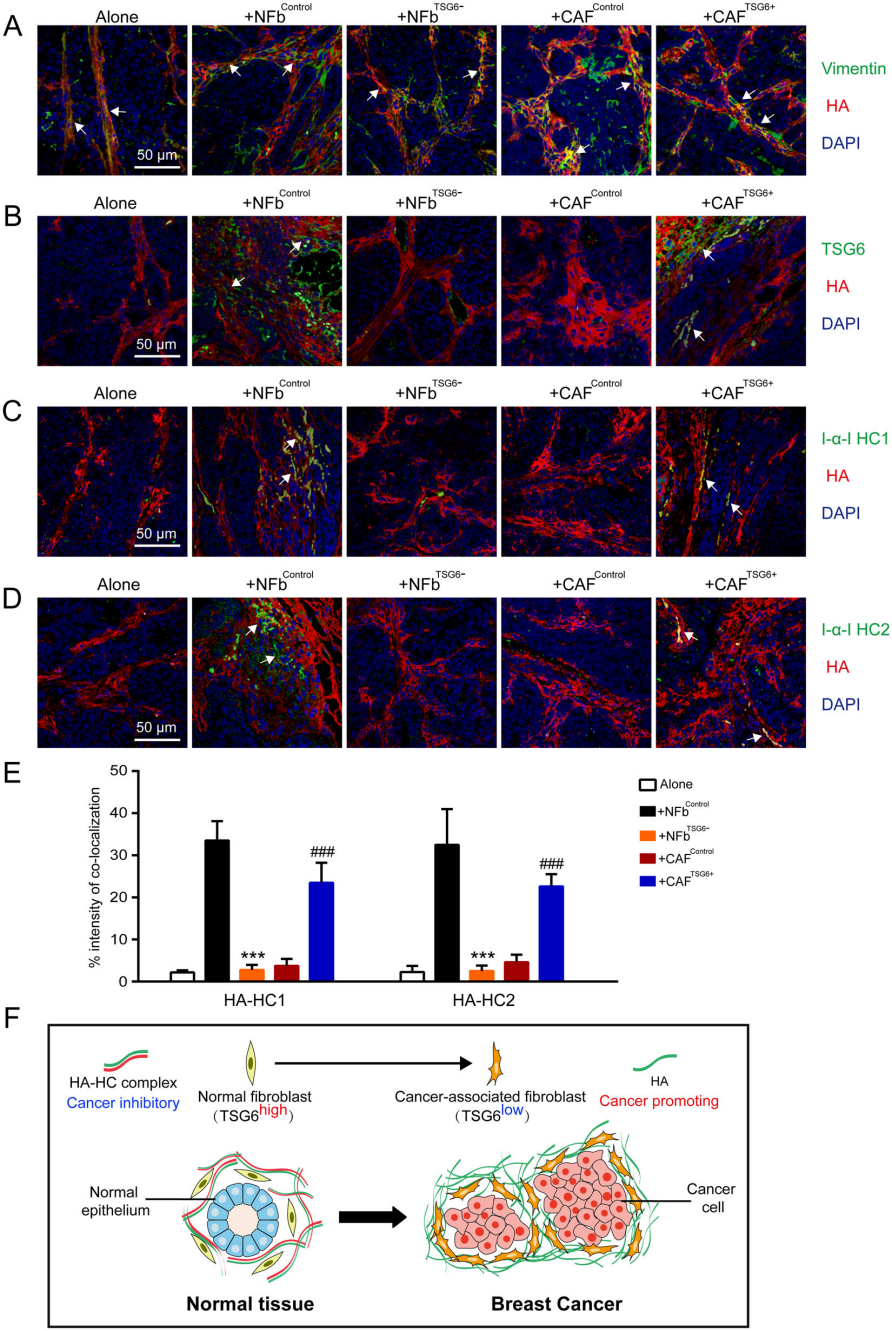
The expression of cross-linked HA in cancer tissues from mouse tumor model. **A** Double staining of HA and vimentin in cancer tissues from different groups. White arrowheads refer to the co-localization of HA (red) and fibroblasts (green, vimentin^+^). **B** Double staining of HA and TSG6 in cancer tissues. White arrows indicate the co-localization of HA (red) and TSG6 (green). **C** Double staining of HA and I-α-I HC1 in cancer tissues. White arrowheads indicate the co-localization of HA (red) and I-α-I HC1 (green). **D** Double staining of HA and I-α-I HC2 in cancer tissues. White arrows indicate the co-localization of HA (red) and I-α-I HC2 (green). **E** The intensity ratio of HA co-localized with I-α-I HCs to total HA was used to determine the levels of HA-HC complexes. Error bars represent mean ± SD values. Statistical analysis was performed using Student’s *t* test. ***p < 0.001 (vs +NFb^Control^), ^###^*p* < 0.001 (vs +CAF^Control^). **F** Graphic depicting a model for the role of HA crossing-linking in CAF-dependent breast cancer malignancy.

We further verified whether the differences of tumor malignancy between groups are primarily due to HA cross-linking. As shown in Fig. 7A, HA was mainly distributed in stoma and co-localized with fibroblasts (vimentin^+^) in all the groups. The levels of TSG6 in NFb^Control^ and CAF^TSG6+^ groups were significantly higher compared to the NFb^TSG6−^ and CAF^Control^ groups, respectively (Fig. 7B). As expected, cancer tissues in the NFb^Control^ and CAF^TSG6+^ groups had more HA-HC1 and HA-HC2 complexes than that in the NFb^TSG6−^ and CAF^Control^ groups (Fig. 7C–E).

As shown in Fig. 7F, when NFbs are activated into CAFs, TSG6 is downregulated. Meanwhile, the levels of cross-linked HA are dramatically decreased, followed by an increment of tumor malignancy.

## Discussion

The promoting role of HA in breast cancer malignancy has been well characterized. Previous studies mainly focused on the quantity and MW of HA in the tumor microenvironment. In this study, we investigated the role of HA in tumor malignancy from a novel perspective, the change of conformation. In the breast cancer microenvironment, we found that cross-linked HA levels were significantly reduced in a CAF-dependent manner, together with an accelerating effect on tumor cell proliferation, migration, and invasion.

HA cross-linking has been identified in physiological and inflammatory conditions and is critical for reproduction and anti-inflammation^19,39,40^. Our study illustrated that cross-linked HA levels were significantly decreased in breast cancer tissues and negatively associated with tumor malignancy. Therefore, we proposed that HA cross-linking might have an inhibitory role in the process of breast cancer.

In many solid tumors, HA was mainly distributed in the stroma and synthesized by CAFs^29–31^. In breast cancer tissues from patients and MMTV-PyMT mice, our data also demonstrated that CAFs were mainly responsible for HA accumulation in cancer stroma. Therefore, we isolated and cultured NFbs and CAFs from mice and breast cancer patients. As expected, the HA level in CAFs was significantly higher than that in NFbs, which is in line with previous studies showing that fibroblasts synthesize more HA when activated into CAFs^41^. However, the cross-linked HA was almost deficient in CAFs. As reported before, suppressing the HA production of CAFs can inhibit tumor malignancy^28^, so we wonder whether altering the levels of cross-linked HA could influence the pro-tumor capability of CAFs on breast cancer. As a covalent complex between HA and HCs from I-α-I, cross-linked HA is catalytically mediated by TSG6^16^. Our experiment found that NFbs and CAFs with different levels of cross-linked HA were obtained through regulating TSG6 expression, whereas the quantities and MW of HA were not changed. As a result, CAFs significantly promoted the malignancy of MMTV tumor cells compared to NFbs in vitro and in vivo. However, the breast cancer malignancy was inhibited when CAFs restored the high level of cross-linked HA, whereas NFbs with a reduced level of cross-linked HA accelerated the malignancy. Our data indicated that the conformational change of HA induced by CAFs was critical in breast cancer malignancy.

TSG6 is a secreted protein implicated in mediating immunomodulatory and beneficial activities of mesenchymal stem or stromal cells^42^. Our study found that TSG6 levels were significantly decreased in CAFs compared to NFbs, which was consistent with the levels of cross-linked HA. Upregulation of TSG6 expression restored HA cross-linking in CAFs, verifying the inhibitory role of cross-linked HA in breast cancer malignancy. Martin et al. reported that TSG6-mediated transfer of I-α-I HC5 to HA facilitated the TGF-β-dependent differentiation of human lung fibroblasts to α-SMA-expressing myofibroblasts, suggesting that TSG6 was involved in lung fibrosis^43^. Although α-SMA is both highly expressed in CAFs and myofibroblasts, the characteristics of CAFs in tumor microenvironment are definitely different from myofibroblasts that induce fibrosis. Therefore, the expression and function of TSG6 may vary in different fibroblasts originated from distinctive tissues and disease microenvironments. Given that TSG6 is a multifunctional molecule, we have no idea whether the regulation of TSG6 has other effects on fibroblasts besides HA cross-linking. To exclude this possibility, the typical pro-tumor cytokines TGF-β, IL-6, EGF, and HGF secreted by CAFs were assessed after the overexpression of TSG6. Our data showed that TSG6 had no significant effect on the levels of CAF-derived pro-tumor cytokines. To further verify whether the tumor-inhibiting effect is induced by cross-linked HA alone, the HA-HC complex was synthesized in vitro. As reported before, the HA-HC complex can inhibit the proliferation of macrophages and the tube formation of human umbilical vein endothelial cells^44,45^. Our study found that the synthesized HA-HC complex suppressed the proliferation and invasion of breast cancer cells in a concentration-dependent manner, confirming the inhibitory role of HA cross-linking in breast cancer malignancy.

Our previous study showed that HA, especially LMW-HA, was abnormally accumulated in the serum and cancer tissues from patients with breast and colorectal cancer and associated with metastasis and prognosis^46,47^. In this study, the promoting role of CAF-derived HA in breast cancer malignancy was suppressed when the conformational state was altered from non-cross-linking to cross-linking. As reported in arthritis, cross-linked HA can resist the adverse effects of HA fragmentation^48^, which may partially explain the inhibitory effects of HA cross-linking on breast cancer malignancy. The detailed underlying mechanisms will be further explored in our future study.

In conclusion, we identified the role of HA in the malignant progression of breast cancer from a novel perspective. Our result demonstrated that HA cross-linking was deficient in breast cancer tissues, with a negative association with tumor malignancy. After restoring HA cross-linking in CAFs by regulating TSG6 expression, breast cancer malignancy was significantly suppressed in vitro and in vivo. Therefore, our study indicated that the deficiency of cross-linked HA in tumor microenvironment was CAF dependent, and restoring HA cross-linking may be a potential strategy for breast cancer treatment.

## Supplementary information

Supplemental Information
