# Supplementary material for: Reduced hyaluronan cross-linking induces breast cancer malignancy in a CAF-dependent manner
Source: Cell Death Dis. 2021 Jun 7;12(6):586. doi: 10.1038/s41419-021-03875-6 (PMC8184848; doi:10.1038/s41419-021-03875-6)
Supplement: Supplementary file 1 — Supplemental Information [file 41419_2021_3875_MOESM1_ESM.docx]

**Supplementary information**

**Supplementary Figures**


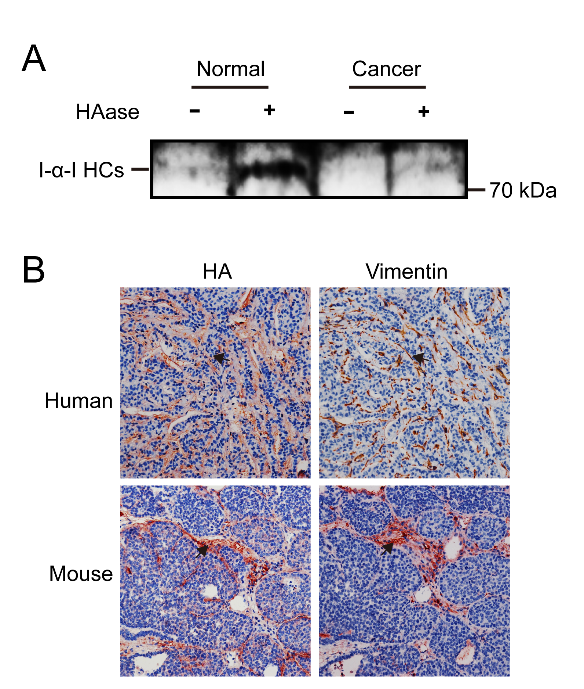


**Fig.S1**

**A: HA-HC complexes in normal mammary glands and breast cancer tissues.** The normal mammary gland and breast cancer tissues were derived from FVB and MMTV-PyMT mice, respectively. The abundance of HA-HC complexes in tissue lysates was detected by western blot using I-α-I antibody with (+) or without (-) the digestion of hyaluronidase (HAase). HAase can release the I-α-I HCs linked to HA, confirming the existence of HA-HC complexes. Compared to breast cancer tissue, the extensive expression of I-α-I HCs in the normal tissue lysate after HAase digestion was detected, suggesting that the levels of HA-HC complexes were significantly decreased in the breast cancer microenvironment.

**B: HA and vimentin expression in cancer tissues from patients with breast cancer and MMTV-PyMT mice.** HA and CAFs (vimentin-positive cells) were stained in serial sections, respectively. As shown, HA (red) was mainly distributed around cancer cell islets. The distribution of CAFs (red) was in accordance with HA deposition, suggesting that HA in the breast cancer microenvironment was mainly derived from CAFs. Black arrows indicate the same positions in serial sections.


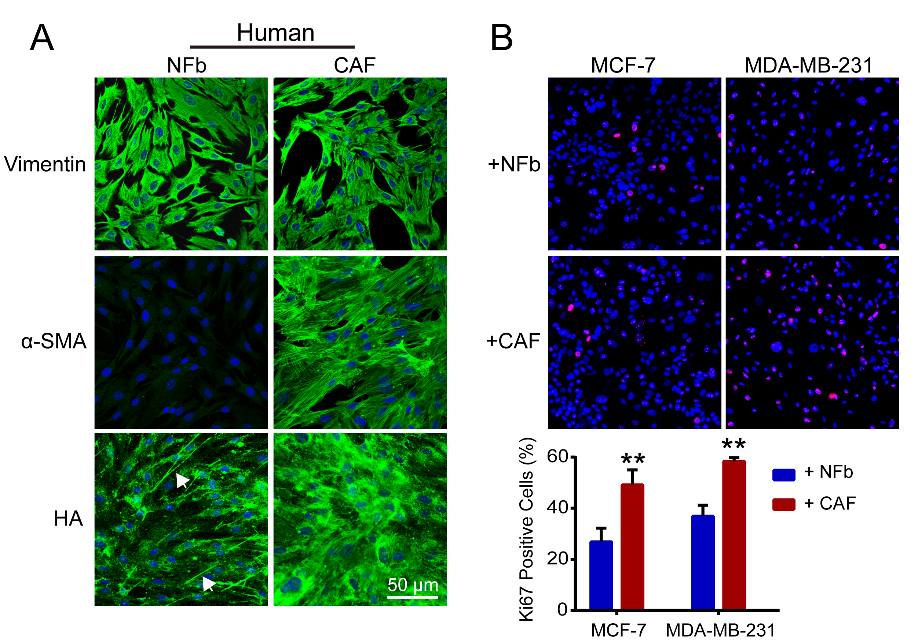


**Fig.S2**

**A: The characteristics of normal fibroblasts (NFbs) and CAFs derived from breast cancer patients.** Vimentin was highly expressed on both NFbs and CAFs, whereas α-SMA was only expressed on CAFs, suggesting that human breast NFbs and CAFs were successfully isolated and cultured. Importantly, cable-like HA (white arrows) was presented on NFbs, whereas CAFs showed no cross-linked HA.

**B: The Ki67 expression of human breast cancer cells co-cultured with fibroblasts in TSA assay.** MCF-7 and MDA-MB-231 cells were separately co-cultured with human NFbs or CAFs. Then Ki67 was detected to evaluate the proliferative ability of breast cancer cells. Compared to NFbs, the Ki67 positive rates of MCF-7 and MDA-MB-231 cells were both significantly increased when co-cultured with CAFs.


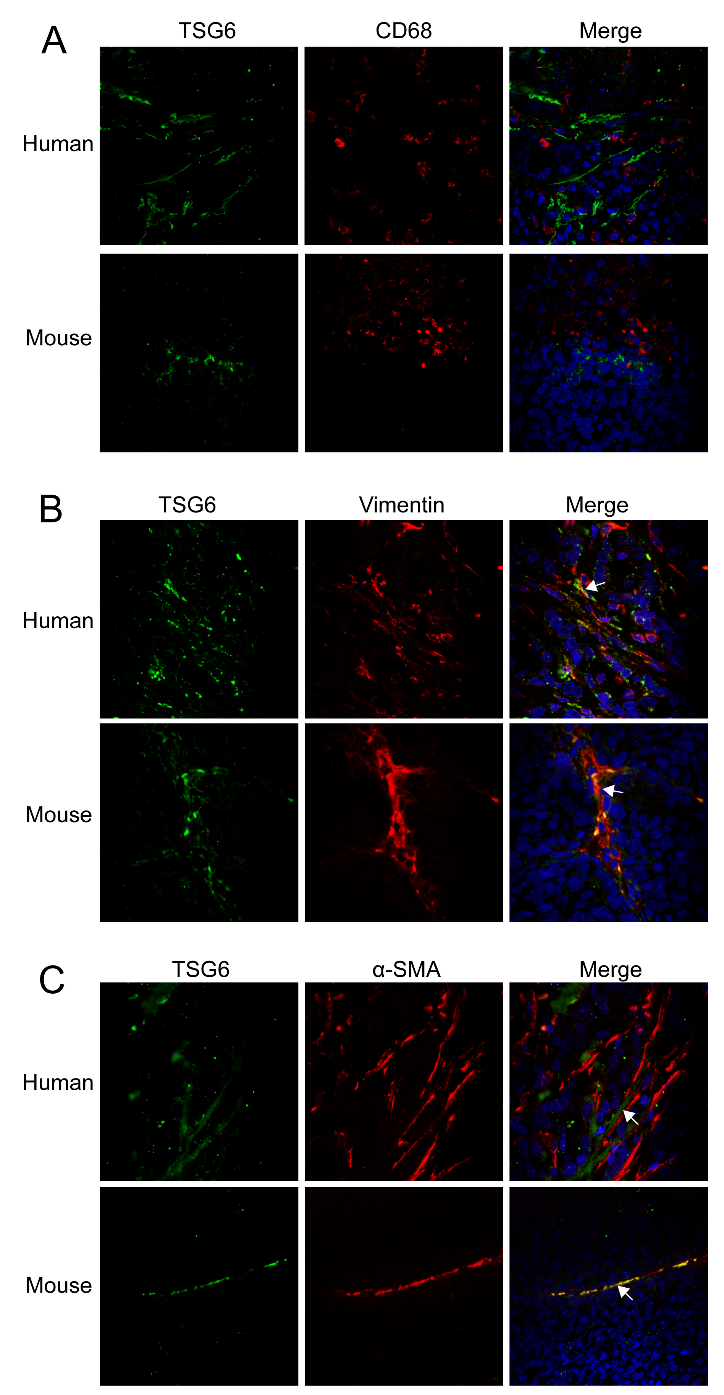


**Fig.S3**

**A: Macrophages and TSG6 in early-staged breast cancer tissues.** CD68 was stained to localize the macrophages in breast cancer tissues. The distribution of macrophages (red) was not in line with TSG6 (green).

**B: The expression of vimentin and TSG6 in** **early-staged breast cancer tissues.** TSG6 (green) and vimentin (red) were co-localized (white arrow) in the breast cancer stroma.

**C: The expression of α-SMA and TSG6 in early-staged breast cancer tissues.** The co-localization (white arrow) of TSG6 (green) and α-SMA (red) was observed in the breast cancer stroma.


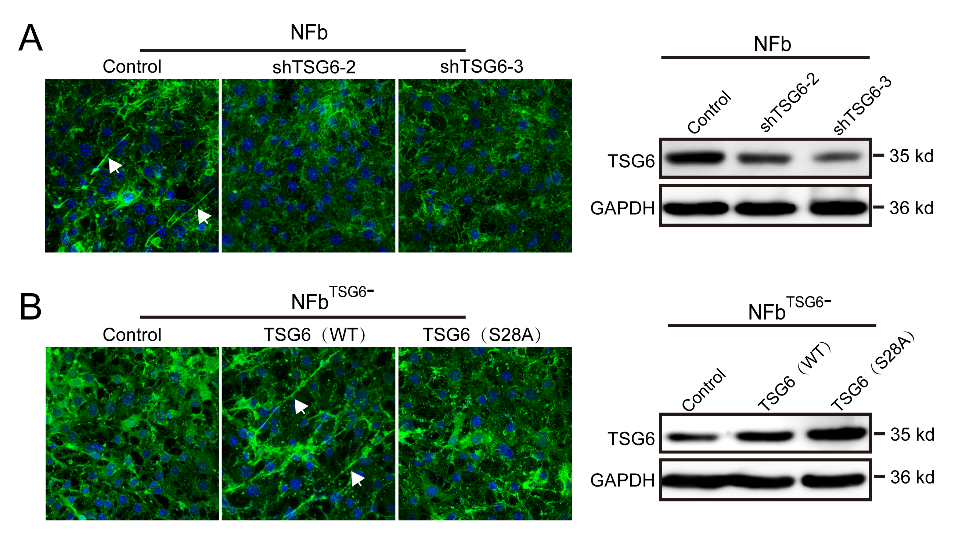


**Fig.S4**

**A: The effect of TSG6 down-regulation on HA cross-linking in NFbs.** NFbs derived from FVB mice were also infected with two other kinds of shTSG6 viruses (No.2 and 3). As shown by Western blot, the protein levels of TSG6 were both down-regulated. Meanwhile, the cross-linked HA levels were also significantly decreased.

**B: The effect of TSG6 overexpression on HA cross-linking in NFb^TSG6-^ cells.** To exclude the off-target effect of siRNA, NFb^TSG6-^ cells were transfected with siRNA-resistant plasmids of wild-type TSG6 or mutant TSG6 (S28A) without catalytic function. After transfection, TSG6 levels were both markedly increased. However, only the wild-type TSG6 could recover the HA cross-linking of NFb^TSG6-^ cells.


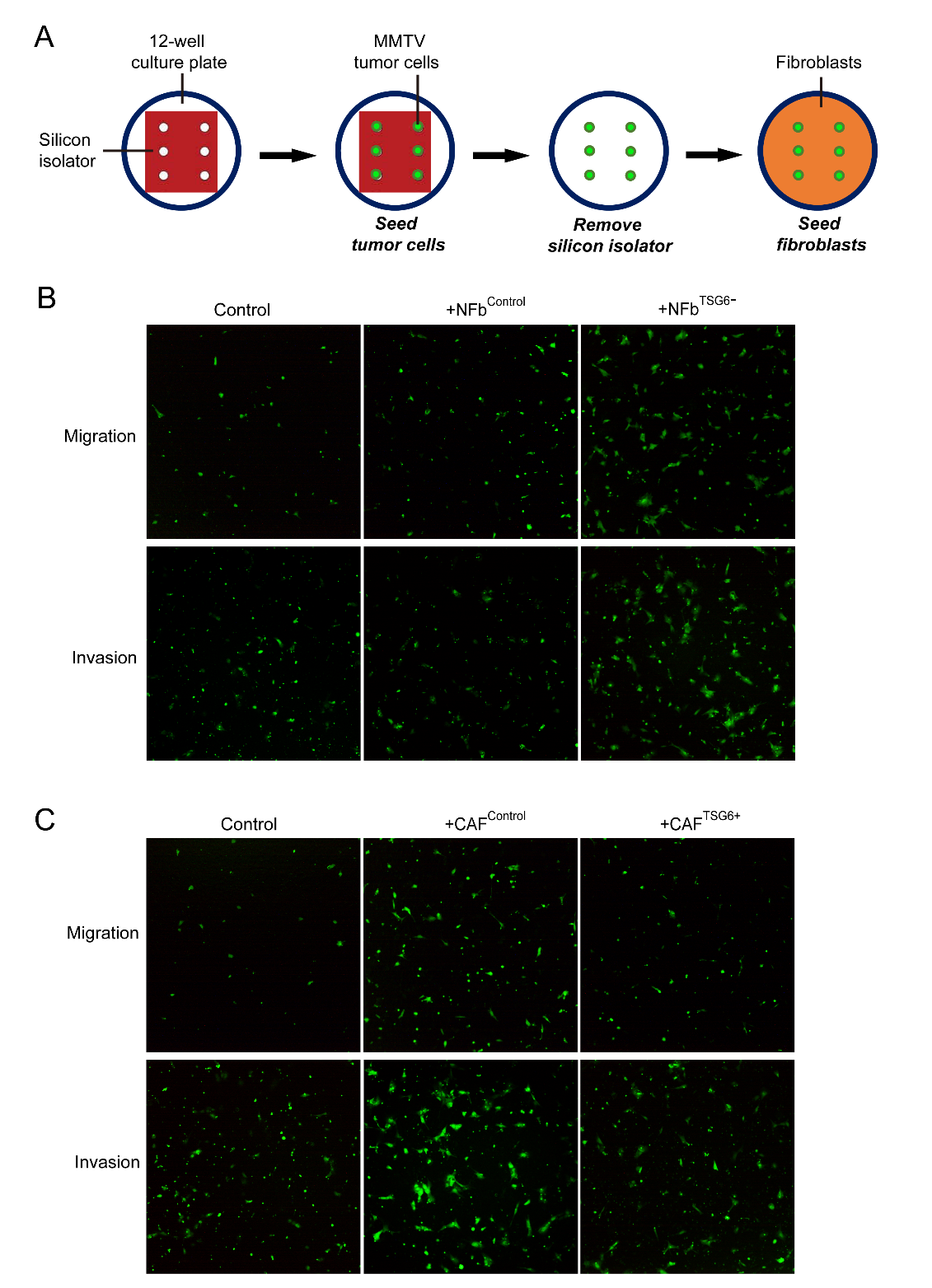


**Fig.S5**

**A: Schematics of the tumor-stromal assay (TSA) process.**

**B: Migration and invasion abilities of MMTV tumor cells co-cultured with different NFbs.** After stained with a cell tracker, MMTV tumor cells (green) were co-cultured with NFb^Control^ or NFb^TSG6-^ cells. As shown, the migration and invasion abilities of MMTV tumor cells were both significantly accelerated when stimulated by NFbs with a low level of cross-linked HA (NFb^TSG6-^ cells).

**C: Migration and invasion abilities of MMTV tumor cells co-cultured with different CAFs.** After stained with a cell tracker, MMTV tumor cells (green) were co-cultured with CAF^Control^ or CAF^TSG6+^ cells. Compared to CAF^Control^ cells, CAFs with a high level of cross-linked HA (CAF^TSG6+^ cells) significantly inhibited the migration and invasion of MMTV tumor cells.


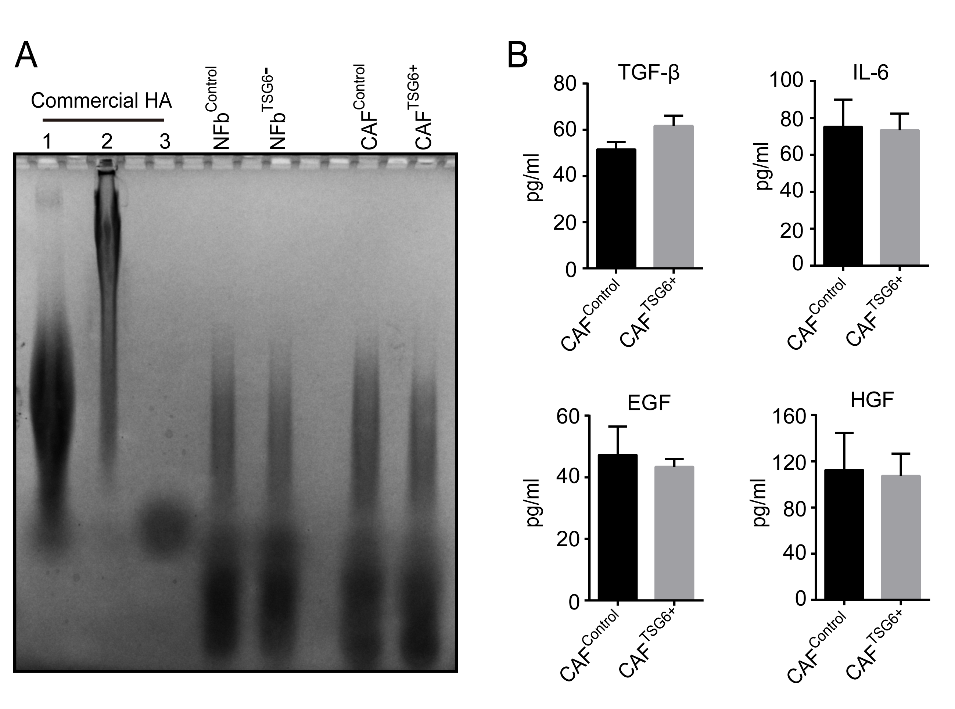


**Fig.S6**

**A: The molecular weight of HA derived from different fibroblasts.** After extraction from the conditional media of NFb^Control^, NFb^TSG6-^, CAF^Control^, and CAF^TSG6+^ cells, HA was analyzed using electrophoresis. Commercial HA was used to indicate the molecular weight. The molecular weight ranges of different HA were as follows. Lane 1: 150-300 kDa, lane 2: 1000-1800 kDa, and lane 3: 10-20 kDa. The molecular weight distributions of HA derived from different fibroblasts were similar.

**B: The levels of cytokines secreted by different CAFs.** The levels of TGF-β, IL-6, EGF, and HGF in the conditional media of CAF^Control^ and CAF^TSG6+^ cells were detected using ELISA. Compared to CAF^Control^ cells, overexpression of TSG6 did not change the levels of pro-tumor cytokines secreted by CAFs significantly.


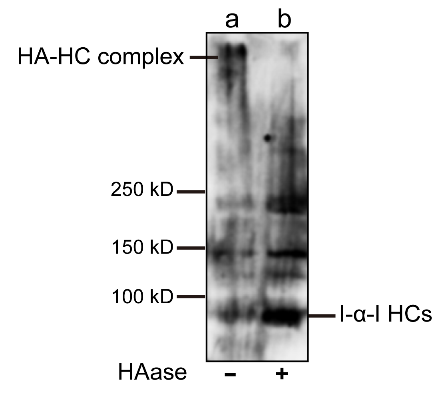


**Fig.S7**

**HA-HC complex synthesized in vitro.** The HA-HC complex was synthesized using HMW-HA, recombinant mouse TSG-6 protein, and mouse serum (source of I-α-I). Then the reaction product was verified by Western blot using I-α-I antibody before (-) and after (+) the digestion of hyaluronidase (HAase). As shown in lane **a**, the band of highest molecular weight indicated the HA-HC complex. After digested with [HAase](http://www.baidu.com/link?url=2O8HHiu5utmrVhvwimXgsO_3Fc3lPDbF7v4wvwPROIz3JnTaAg0bL3kp3kgBGuAZ4ZDAmm9xsP0w9-FnN2G5dE6C6ekjWvqbgjOCmoDwkLe), I-α-I HCs was released from the HA-HC complex, resulting in the significantly decreased HA-HC complex band and increased I-α-I HCs band (lane **b)**. This data indicated that the HA-HC complex was successfully synthesized.


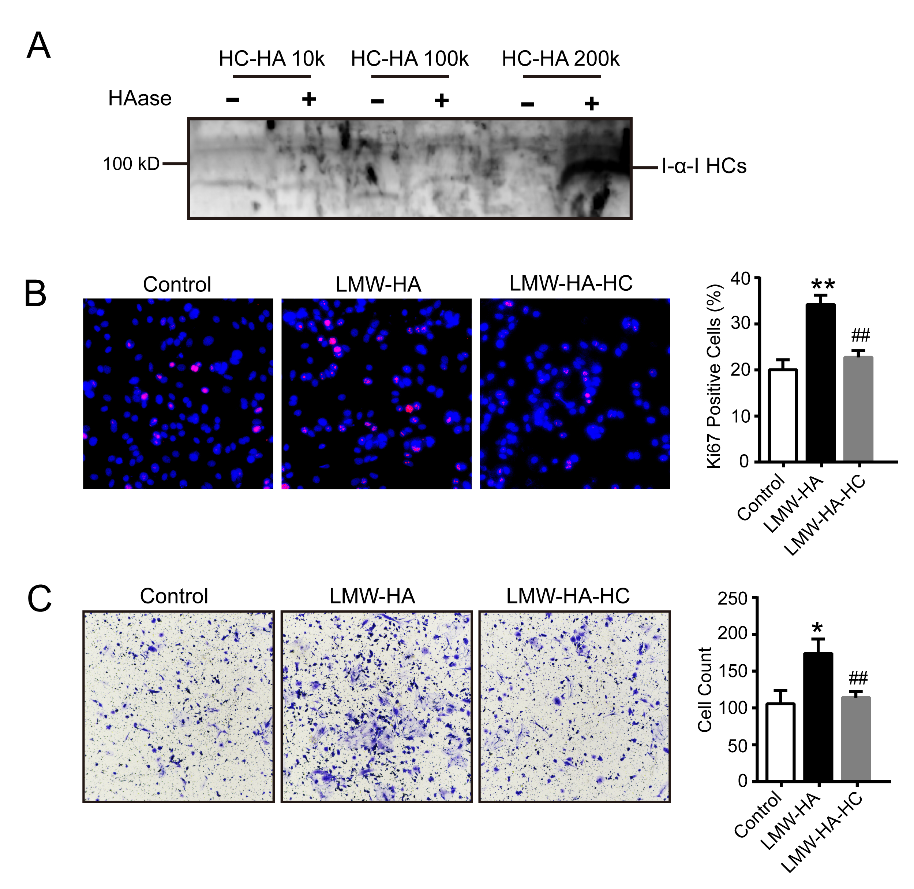


**Fig.S8**

**A: Verification of LMW-HA-HC complex using Western blot.** Three kinds of LMW-HA were used to synthesize LMW-HA-HC complexes. The weight-average molecular weight (Mw) was 10 kDa, 100 kDa, and 200 kDa, respectively. Then the LMW-HA-HC complexes were detected by I-α-I antibody before and after the digestion of HAase. After digested by HAase, the level of I-α-I HCs was only significantly increased in LMW-HA-HC complex synthesized by 200 kDa HA, indicating that 200 kDa HA can form the LMW-HA-HC complex.

**B: The effect of LMW-HA-HC complex on the proliferation of MMTV tumor cells.** After stimulated by LMW-HA (200 kDa), the Ki67 positive cells were markedly increased. In contrast to LMW-HA, LMW-HA-HC complex inhibited the proliferative ability of tumor cells. Statistical analysis was performed using Student's t-test. ** *p*<0.01 (* vs Control), ^##^ *p*<0.01 (^#^ vs LMW-HA).

**C: The effect of LMW-HA-HC complex on the invasion of MMTV tumor cells.** LMW-HA promoted the invasion of MMTV tumor cells. However, the invasive ability of tumor cells was suppressed by LMW-HA-HC complex compared to LMW-HA (200 kDa). Statistical analysis was performed using Student's t-test. * *p*<0.05 (* vs Control), ^##^ *p*<0.01 (^#^ vs LMW-HA).

**Supplementary Table**

**Table.S1 Clinical characteristics of study subjects.**

| **Clinical characteristics** | **Breast cancer patients (n = 16)** |
| --- | --- |
| **Histological type** | Invasive ductal carcinoma (n = 16) |
| **Histological grade** | 1 (Nottingham Score = 3~5, n = 7)  3 (Nottingham Score = 8 or 9, n = 9) |
| **Molecular subtypes** | Luminal A: n = 2  Luminal B: n = 9  HER2-enriched: n = 3  Triple-negative phenotype: n = 2 |
| **TNM stage** | Stage Ⅰ: n = 7  Stage Ⅲ: n = 9 |
| **Average age** | 63 |
